# A Case of Acute Rheumatism—Metastasis to the Heart—Maltreatment—Death

**Published:** 1847-06

**Authors:** W. Wadsworth

**Affiliations:** Racine, Wisconsin Territory


### ARTICLE VI.

*A Case of Acute Rheumatism—Metastasis to the Heart—Mal-Treatment—Death.* By W. Wadsworth, M. D., of Racine, Wisconsin Territory.

Mr. B. P., Æt. 32, who had pursued a laborious employment, that of brick-making, was attacked about three months since* by acute rheumatism, confined chiefly to the left shoulder and arm. While yet about the streets, his arm suspended in a sling, he stepped into the office of a Botanic German Physician, who professes extraordinary skill (by looking at the urine!!) in diagnosticating diseases as well as curing them. An examination of his case led to the remarkable discovery that his shoulder was out of joint. An attempt was immediately made to reduce it, which, from the inflamed and tender state of the member, was attended with extreme suffering, fully satisfying the patient of the difficulty, and inspiring no little respect for the the man whose far-reaching intellect had unravelled his complicated and, up to this time, unsuspected malady. Thus deceived at the outset, he more readily became his dupe and, as the sequel will unfortunately prove, his victim.

In the course of the disease, and, as nearly as can be ascertained, at an early period, there occurred a metastasis or translation of the inflammation to the heart. This alarming event, for which an energetic and well-timed treatment

* The case occurred March 20th, 1846.

is required, was wholly overlooked, and, what is worse, maltreated by stimulants and tonics, increasing the force of the circulation, confirming and riveting, as with fetters of iron, this dangerous but not obscure disease. The consequences of this practice were soon apparent, outraging pathological laws as well as therapeutic indications. For as the circulation was unduly augmented and raised above the natural standard, and urged on by the irritated, inflamed, and laboring heart, nature struggling to find some avenue through which to offer mitigation, permitted, or rather forced the blood to gush freely forth from the nose. A temporary calm was thus induced. But stimulants reapplied, the same results were again and again witnessed, till palor and exhaustion coming on, nature, in her unequal struggle, gave signs that all was lost! At this time, a medical man of some experience in these cases, by the urgent request of a friend, saw him, pointed out the disease and its probable consequences; but his opinion was wholly disregarded, so strongly were they entrenched by the loud and confident assurances, of their attendant, of a successful issue—a speedy and complete restoration; and these falsehoods, backed up and sustained by another charlatan who, in consultation with him, declared "that whatever might be the difficulty, nothing ailed the heart."

In the meantime the disease made rapid progress. The impulse of the heart was not only felt at each pulsation in the chest, but through the entire brain, accompanied by paroxysms of laborious breathing, intense anxiety, and unmitigated agony.

At this late period I, with my friend Dr. Smith, was called in. The history, and a careful examination of the case, clearly revealed that the curative indications had passed; that organic disease had seized on the citadel of life, and the resources of our noble art could alone mitigate urgent symptoms, and smooth the rugged pathway to the grave. He shortly expired.

A post mortem examination was obtained, and the entire faculty of the town was present. The body was considerably emaciated. The left side, over the region of the heart, was more prominent than the right. On opening the chest, adhesions were extensively present in the left side. The pericardium was inflamed, containing six ounces of bloody serum. The endocardium, exhibited still more striking evidence of inflammatory action. Lymph was abundantly thrown out and found in and about the large vessels of the heart, the valves thickened, and other structures enlarged. The muscular structure of the organ in some portions blackened and nearly disorganized. A more marked case of morbid anatomy is rarely seen.

He who so long and stupidly treated this case, and had declared there was no disease of the heart, was also present at the examination. He was, however, opposed to the examination, and did what he could to arrest it, as he and his friends had assumed that the change which had taken place in his management a few days before death, was the cause of that unfortunate result. They were congratulating themselves on the old, but in this case mistaken, saying, "that the dead tell no tales." And why did he shrink from this investigation which was to reveal the truth and facts of a case with which he had been so long conversant, if he did not begin to suspect that his gross ignorance and stupidity would see the light? The light they did see; and while the morbid structure of the heart was pointed out to him, he stood amazed and confounded. He must have seen with what recklessness he had trifled with the high and dearest interests of humanity, and if he had any moral feeling left must have passed on himself a severe and terrible condemnation. Will this solemn opportunity be lost upon him? Most undoubtedly it will, for what can be hoped from one whose whole system is founded in duplicity and fraud. But let him not complain if the veil is occasionally drawn aside and its fearful consequences exposed to the world.'

